# Supplementary material for: Structure of SARS-CoV-2 ORF8, a rapidly evolving immune evasion protein
Source: Proc Natl Acad Sci U S A. 2020 Dec 23;118(2):e2021785118. doi: 10.1073/pnas.2021785118 (PMC7812859; doi:10.1073/pnas.2021785118)
Supplement: Supplementary File [file pnas.2021785118.sapp.pdf]

PNAS
www.pnas.org

Supplementary Information for
Structure of SARS-CoV-2 ORF8, a rapidly evolving immune evasion protein

Thomas G. Flower, Cosmo Z. Buffalo, Richard M. Hooy, Marc Allaire, Xuefeng Ren and James H. Hurley

James H. Hurley
Email: jimhurley@berkeley.edu

This PDF file includes:

Table S1

Table S1. Data collection and refinement statistics

	SARS-CoV-2 ORF8 (PDB 7JTL)	
**Data collection**		
	Native	SeMet
Space group	$P4_12_12$	$C2_1$
Cell dimensions		
a, *b*, *c* (Å)	44.3 44.3 264.1	69.5 69.5 139.6
α, β, γ (°)	90, 90, 90	90, 104, 90
Resolution (Å)	43.65-2.04 (2.11-2.04)	48.44-2.98 (3.16-2.98)
R_{pim}	0.032 (0.555)	0.037 (0.600)
$I / \sigma I$	14.92 (1.20)	12.3 (1.7)
Completeness (%)	97.8 (90.2)	99.4 (99.4)
Redundancy	10.0 (7.6)	5.6 (5.9)
**Refinement**		
Resolution (Å)	43.65-2.04	
No. reflections	17005 (1399)	
R_{work} / R_{free} (%)	22.0 (35.2)/ 26.4 (38.4)	
No. atoms		
Protein	1609	
Water	201	
B-factors		
Protein	45.4	
Water	44.3	
R.m.s. deviations		
Bond lengths (Å)	0.008	
Bond angles (°)	1.00	

Values in parentheses are for highest-resolution shell. Redundancy for SeMet data is shown for I+ and I- separately
